# Identifying locally actionable strategies to increase participant acceptability and feasibility to participate in Phase I cancer clinical trials

**DOI:** 10.1111/hex.13920

**Published:** 2023-12-02

**Authors:** Stephanie A. S. Staras, Easton N. Wollney, Lisa E. Emerson, Natalie Silver, Peter T. Dziegielewski, Marta D. Hansen, Gabriela Sanchez, Dalila D'Ingeo, Versie Johnson‐Mallard, Rolf Renne, Kristianna Fredenburg, Michael Gutter, Kendra Zamojski, Carla Vandeweerd, Carma L. Bylund

**Affiliations:** ^1^ Department of Health Outcomes and Biomedical Informatics University of Florida College of Medicine Gainesville Florida USA; ^2^ Department of Microbiology and Cell Science, Institute of Food and Agricultural Sciences University of Florida Gainesville Florida USA; ^3^ Department of Surgery Cleveland Clinic Cleveland Ohio USA; ^4^ Department of Otolaryngology University of Florida College of Medicine Gainesville Florida USA; ^5^ Center for Nursing Research College of Nursing, Kent State Kent Ohio USA; ^6^ Department of Molecular Genetics and Microbiology University of Florida College of Medicine Gainesville Florida USA; ^7^ Department of Pathology University of Florida College of Medicine Gainesville Florida USA; ^8^ Department of Family, Youth and Community Sciences, Institute of Food and Agricultural Sciences University of Florida Gainesville Florida USA

**Keywords:** cancer, community oncology, patient education, Phase I, underrepresentation

## Abstract

**Background:**

Recruitment of cancer clinical trial (CCT) participants, especially participants representing the diversity of the US population, is necessary to create successful medications and a continual challenge. These challenges are amplified in Phase I cancer trials that focus on evaluating the safety of new treatments and are the gateway to treatment development. In preparation for recruitment to a Phase I recurrent head and neck cancer (HNC) trial, we assessed perceived barriers to participation or referral and suggestions for recruitment among people with HNC and community physicians (oncologist, otolaryngologist or surgeon).

**Methods:**

Between December 2020 and February 2022, we conducted a qualitative needs assessment via semistructured interviews with a race and ethnicity‐stratified sample of people with HNC (*n* = 30: 12 non‐Hispanic White, 9 non‐Hispanic African American, 8 Hispanic and 1 non‐Hispanic Pacific Islander) and community physicians (*n* = 16) within the University of Florida Health Cancer Center catchment area. Interviews were analyzed using a qualitative content analysis approach to describe perspectives and identify relevant themes.

**Results:**

People with HNC reported thematic barriers included: concerns about side effects, safety and efficacy; lack of knowledge and systemic and environmental obstacles. Physicians identified thematic barriers of limited physician knowledge; clinic and physician barriers and structural barriers. People with HNC and physicians recommended themes included: improved patient education, dissemination of trial information and interpersonal communication between community physicians and CCT staff.

**Conclusions:**

The themes identified by people with HNC and community physicians are consistent with research efforts and recommendations on how to increase the participation of people from minoritized populations in CCTs. This community needs assessment provides direction on the selection of strategies to increase CCT participation and referral.

**Patient or Public Contribution:**

This study focused on people with HNC and community physicians' lived experience and their interpretations of how they would consider a future Phase I clinical trial. In addition to our qualitative data reflecting community voices, a community member reviewed the draft interview guide before data collection and both people with HNC and physicians aided interpretation of the findings.

## 1 INTRODUCTION

Twenty percent of cancer treatment trials fail due to low patient recruitment. Only 8% of patients with cancer participate in a cancer clinical trial (CCCT) due to structural barriers, clinical barriers, physician attitudes and patient attitudes.^1^, ^2^, ^3^, ^4^ Minoritized populations, including Black, Hispanic and rural patients with cancer, are even more underrepresented in CCCTs (approximately 4% participating) and especially in Phase I CCCTs and in the COVID‐19 era.^5^, ^6^, ^7^, ^8^, ^9^ The consequences of this inequitable recruitment are that many CCCTs and the treatments they verify fail to reflect the US population.^10^ It is imperative to understand the barriers to enroling diverse populations in CCCTs, especially Phase I trials, to increase the generalizability of the results.

Community medical and radiation oncologists are key to the success of CCCTs because they are the gatekeepers between community patients and CCCTs.^2^, ^11^ Barriers to clinical trial referral include increased workloads associated with identifying trials and discussing them with patients, financial losses stemming from referring the patient to another centre, lack of knowledge about specific study details and lack of applicability of CCT findings to community practice.^12^, ^13^, ^14^ Researchers have begun to identify facilitators to CCT recruitment in collaboration with community physicians including (1) creating a research community between community physicians and researchers, (2) securing resources to support clinical recruitment without interfering with clinical care, (3) research designs that account for patient concerns regarding feasibility and potential benefit, (4) acknowledging the community physicians' professional roles as a patient advocate and (5) recruitment strategies that endure patient trust, show benefit to patients, and follow an effective system,^15^ but to date little is known about barriers and facilitators in targeting recruitment of minoritized populations. A greater understanding of community specialty physicians' perceived barriers and recommendations on how to improve clinical trial recruitment of underrepresented populations to Phase I CCTs is an important step to equitably improving cancer treatment.

Across studies, approximately 55% of patients offered CCT participation agree to participate.^16^ The main reasons for nonparticipation include the desire for alternative treatment (25%), not being interested in trial participation (20%), passive refusals by not returning to the clinic (8%), fear of side effects (8%), financial concerns (7%) or dislike of being in an experiment (7%).^16^ For Phase I cancer CCTs, patient motivators for participating are the hope of therapeutic benefit (primary) and advancing scientific knowledge for others (secondary).^17^ However, after recruitment, Phase I CCT participants expressed unmet expectations of tumour response and logistical barriers related to travel.^18^ Increased understanding of underrepresented people with HNC preferences for considering Phase I CCT participation, especially when not currently contemplating enrolment, would aid the creation of informative and acceptable recruitment strategies.

In preparation for enroling people with recurrent head and neck cancer (HNC) into a first‐in‐human Phase I therapeutic CCT using personalized immunotherapy, we wanted to understand the perceived barriers to this specific trial for people with HNC (group 1) participating and community physician (group 2) referring patients. We had two distinct research questions for each group. For people with HNC:

1. What factors do people with HNC think should be considered if deciding to participate in a Phase I CCT?
2. What strategies can increase the number of people who participate in the planned Phase I CCT?

For community physicians:

1. What barriers exist to referring patients to CCTs? and
2. What recommendations do physicians have for improving cancer clinical trial referrals?

## 2 METHODS

### 2.1 Overview of approach

To answer the research questions above, we conducted 30‐min qualitative, structured interviews with two populations: (1) people who had previously been diagnosed with HNC (target 30 for thematic saturation), and (2) community physicians who treat people with HNC; specifically, medical oncologists, radiation oncologists and otolaryngologists (target 15–20 for thematic saturation). We chose this design because we wanted an in‐depth understanding of the acceptability and feasibility of referring or participating in a Phase I HNC therapeutic vaccine trial among people with HNC and HNC community physicians. We relied on a 2022 systematic review of qualitative studies that indicated that 9–17 interviews are generally necessary to reach saturation.^19^ The consent and all study processes were approved by the University of Florida Institutional Review Board. All participants were sent a written description of the consent, provided a brief description during the phone call, and agreed to participate under a waiver of documentation of consent. Interviews were conducted over the phone, audio recorded, and professionally transcribed for analysis. Participants received $50 electronic gift cards for completing the interview.

#### 2.1.1 Procedures and population

To maximize the likelihood that our findings would impact the Phase I therapeutic CCT being acceptable to the community,^20^ we assembled a multidisciplinary team of basic scientists, surgeons, behavioural interventions and clinical care, community‐based Institute of Food and Agricultural Science Extension agents, communication experts, a patient representative and implementation scientists. This group provided feedback on all recruitment procedures and the interview guides.

People with HNC were recruited through advertisements (posted and distributed flyers, press releases in county newspapers, Facebook postings, contact with community cancer support groups) and direct contacts (referral from physicians who participated in the interviews by providing patients with a study flyer, and University of Florida HNC surgeon calls to patients). People with HNC who were interested in participating either contacted the study staff directly or were connected to the study staff by the University of Florida surgeon. Study staff screened interested participants for eligibility: diagnosis with HNC cancer, residing in Florida and meeting race/ethnicity targets.

We aimed for a stratified sample by race/ethnicity among people with HNC with a goal of 10 non‐Hispanic White, 10 non‐Hispanic Black and 10 Hispanic for group‐specific saturation. Advertising and referral from physicians who participated in the interviews resulted in the recruitment of only White people with HNC. Thus, after we had recruited 12 White people with HNC we stopped advertising, excluded additional interested White people with HNC, and focused recruitment on direct contact by the University of Florida HNC surgeon to non‐Hispanic Black and Hispanic people with HNC. Patient interviews were conducted between 21 May 2021 and 17 February 2022.

Community physicians were recruited via two direct contact strategies. First, two Institute of Food and Agricultural Science Extension agents who are embedded in the community and provide a variety of services aimed at improving community health including agricultural support, educational courses, and youth development programmes, collated HNC community physicians within their covered counties (Jefferson, Leon and Citrus), mailed an invitation letter that included a request to call study staff to set up an interview time. Second, university HNC surgeons and co‐authors (N. S. and P. T. D.) sent personal and individual text messages to community physicians who had referred patients to or consulted with University of Florida HNC specialists. Once physicians replied to the text message, the HNC surgeons provided the contact information to a study team member who scheduled interviews, screened physicians for eligibility (treating at least one person with HNC cancer in the past year) and conducted the interview. Physician interviews were conducted between 11 December 2020 and 12 February 2021.

The original structured interview guide was drafted with room for probes and/or follow‐ups as needed. Once drafted, questions were reviewed by all team members. The interview questions were based on Unger's model pathway for clinical trial enrolment and focused on structural, clinical, physician attitudinal and patient attitudinal barriers.^2^, ^3^ Questions for people with HNC included: (1) prior experience with CCT, (2) most important considerations for friends when considering participating in a Phase I therapeutic vaccine trial to treat HNC cancer (described as ‘will be among a small number of participants to make sure the vaccine is safe’) at the University of Florida, (3) preferred person to invite you to participate in such a trial, (4) concerns about travelling to Gainesville, Florida, (5) suggestions on how to increase participation and (6) demographics. Physician questions included: (1) fit of clinical trial referral into the scope of practice, (2) considerations when recommending a patient to a trial, (3) obstacles and facilitators to patients participating in trials, (4) suggestions for proposed strategies to let physicians and patients know about trials and (5) demographics. Probes were utilized to clarify participant perspectives, experiences and/or to solicit more information about views expressed.

After the first interviews (six people with HNC and eight physicians), four team members with expertise in implementation science, the vaccine clinical trial and immunology reviewed the interview recordings following a process review guide to decrease interview length, revise questions to help participants understand the desired constructs, add probes and add questions for any missing construct. Suggested changes in the interview guide were reviewed and agreed upon by the entire research team. Important changes included: shortening the physician interviews, shifting the focus of the majority of the questions for people with HNC from referring to thoughts about themselves to ‘a friend’ since many patients had difficulty viewing themselves as eligible for clinical trials, and enhancing the explanation of Phase I trials to people with HNC.

### 2.2 Data analysis

To analyze and describe findings, we conducted a qualitative content analysis (QCA) for the people with HNC and physician interview data,^21^, ^22^, ^23^ which is ‘a systematic classification process of coding and identifying themes or patterns’.^21^ ^,p.^ ^1278^ Specifically, we used conventional QCA approaches, which include deriving codes from the data during analysis and collapsing codes into larger groups based on similarities.^21^, ^24^, ^25^ Data for people with HNC and physicians were coded separately using similar processes described below. Two codebooks were established (one for people with HNC and one for physician data) and codes were iteratively refined throughout the coding process to describe and define categories/subcategories and ensure rigour through documentation of the process.^21^, ^26^, ^27^ All qualitative data for interviews was managed with standard qualitative management software: ATLAS.ti for of data from people with HNC and InVivo for physician data.

#### 2.2.1 Interview data for people with HNC

Two coders (E. N. W., L. E. E.) began by familiarizing themselves with data and creating initial codes. After going back through the transcripts, one author (E. N. W.) collapsed the initial codes (*n* = 23) into two categories and seven subcategories based on similarities,^28^ with ongoing and regular feedback from additional authors throughout the analysis process (C. L. B., S. A. S. S., L. E. E.) to increase validity and trustworthiness of results.^23^, ^26^ To further ensure rigour, one author (L. E. E.) performed code‐checking and two authors (C. L. B., S. A. S. S.) assessed for face validity of findings as experts with knowledge of health outcomes, communication, and clinical trial participation. Transcripts were segmented before coding to account for race/ethnicity to see if there were any differences in experiences among people with HNC. In addition, three closed‐ended questions were tallied quantitatively: individuals’ personal experience with trials, who they would want to invite them to a trial, and whether they had concerns about coming to our cancer centre for a trial.

#### 2.2.2 Physician interview data

Two coders (C. L. B., V. J.‐M.) began by reading the physicians’ answers to interview questions. One author (C. L. B.) developed an initial set of codes for each of the research questions (*n* = 22) based on the data and subsequently collapsed those, resulting in two categories and nine subcategories. Another author (V. J.‐M.) validated this coding by reading through all physician interview data and adding examples. A third author (D. D.) provided a final verification check to ensure validity.

## 3 RESULTS

Overall, 30 people with HNC and 16 physicians participated (Table 1). People with HNC were an average age of 62 years old, represented approximately equal percentages of men and women and nearly equally represented the targeted racial/ethnic groups. Physicians saw on average 76 HNC patients each year were nearly all White males, were divided between clinical specialties, and were primarily from private practice.

**Table 1:** Demographics of participating people with HNC and community physicians who treat HNC.^a^

	People with HNC % (*n*)	Physician % (*n*)
Total participants	30	16
Age in years	*M* = 61.6, range 37–83	
Years in practice		*M* = 21.9, range 3–38
Number of HNC patients seen/year		*M* = 76, range 7–250
Gender		
Female	46.7% (14)	6.2% (1)
Male	53.3% (16)	93.8% (15)
Race and ethnicity		
White (non‐Hispanic/Latino)	40% (12)	100% (16)^b^
Black or African American (non‐Hispanic)	30% (9)	0% (0)
Hispanic (any race)	27% (8)	0% (0)
Native Hawaiian/Pacific Islander (non‐Hispanic)	3.3% (1)	0% (0)
Clinical specialty		
Radiation oncology		43.7% (7)
Medical oncology		18.8% (3)
Otolaryngology		25% (4)
Surgery		12.5% (2)
Clinical setting		
Private practice		87.5% (14)
Academic setting		12.5% (2)

Abbreviation: HNC, head and neck cancer.

^a^ Presented as percentage and *N* size [% (*n*)], except for age, years in practice and the number of HNC patients seen per year.

^b^ Ethnicity unavailable for one oncologist.

Recruitment methods differed in effectiveness by group. Among the people with HNC, we recruited only people who identify as White from traditional methods: advertising (*n* = 4) and direct contact through community connections (*n* = 5). After which point, we switched to recruiting only via direct contact from participating community physicians (*n* = 2 White), or a HNC surgeon (*n* = 1 total; eight Hispanic, nine non‐Hispanic African American, one non‐Hispanic White and one non‐Hispanic Pacific Islander). Among physicians, only one responded to the mailed invitation and 15 responded to personal text messages from HNC surgeons.

Results from people with HNC and physician interviews are presented by categories (subheadings, bolded) and subcategories (presented in‐text in italics).^25^ Quotations from participant interviews are used as examples and are attributed to participants’ study identification numbers (e.g., ‘P#’ for patient quotes and ‘C#’ for physician quotes).

### 3.1 People with HNC‐identified factors impacting CCT participation

People with HNC described factors they thought should be considered if deciding to participate in a Phase I cancer CCT including *side effect or safety concerns, efficacy of the treatment, a lack of knowledge* about clinical trials and *systemic and environmental factors* (Table 2). These factors were mostly described as potential barriers to enrolment.

**Table 2:** Descriptions of barriers related to CCT participation and referral.

Categories and subcategories	Description
Participation barriers reported by people with HNC	
Concerns about side effects and safety	Concerns about the safety of the treatment or participating in the trial, including any negative or unknown side treatment effects.
Concerns about efficacy	For example, does it work? Will it work? How well does it work compared to traditional treatments?
Lack of knowledge	Having little or no knowledge or information about the treatment or trial; makes decision‐making about participation more challenging. This may make participation in CCTs seem ‘scarier’ to patients.
Systemic and environmental barriers	Includes concerns about the inconvenience to the participant, driving/transportation distance, and if the potential cost is covered by health insurance.
Referral barriers reported by physicians	
Patient barriers	Patient age, distrust of trials, lack of self‐advocacy, lack of resources, and views on treatment efficacy of Phase I CCT (e.g., Phase I vs. Phase IV trials).
Clinical barriers	Barriers regarding the type of medical practice (e.g., private practice or academic medicine) and potential lack of revenue for referring physicians, and lengthy/time‐consuming paperwork.
Physician barriers	Physician's lack of knowledge about ongoing trials; physician's ability to understand trial information.
Structural barriers	Barriers related to transportation options, logistics/distance, time and health insurance.

Abbreviations: CCT, cancer clinical trial; HNC, head and neck cancer.

People with HNC wanted to know about any *side effect or safety concerns* associated with the CCT treatment, particularly as Phase I CCTs were identified by many participants as being new and/or novel. Participants expressed concerns that newer treatments may not be safe or could have a greater likelihood of producing negative or unknown side effects: ‘Its results are basically unknown if you ask me because it's brand new.… Some type of negative side effects [may be] unknown because it's a new experimental drug’ (P09).

Similarly, people with HNC expressed concerns about the *efficacy of the treatment* (i.e., does it work? Will it work?), especially compared to standard treatment options. Participants sometimes viewed decreased treatment efficacy as inherent to clinical trial participation: ‘I mean, potentially it wouldn't be as effective… I don't think that if you participated in a trial, you would necessarily have anything other than ineffective or less effective treatment than doing no treatment at all’ (P10). Some also viewed participation in clinical trials as negatively impacting their ideal treatment schedule and further increasing time spent in treatment: ‘maybe your body does not react to the treatment the way that they're expecting it to… I mean, you could be wasting some of the time that you could have been spending going through a proven treatment track instead’ (P03).

People with HNC sometimes expressed a *lack of knowledge* or lack of information about Phase I CCTs as a potential barrier to enrolment (e.g., ‘I know very little about medical [topics]’ P30). They described needing more information about how Phase 1 CCTs work and/or more information about the trial treatment being offered before agreeing or declining to participate. As one participant explained, ‘You don't know what's going to happen. You don't know what the drug is going to do. You don't know what the procedure's going to do. I think it's scary’ (P02).

Lastly, *systemic and environmental factors* included concerns about time requirements, distance participants would have to travel, transportation to and from the treatment and any costs they would have to cover themselves. Some described travel barriers such as their inability to drive: ‘It's a little far for me to go to. I don't have nobody to drive me there’ (P18); others also described travel as a barrier, though this was due to preference and convenience, and not inability to do so: ‘There may be some inconveniences about participating, [like] having to travel to the central site’ (P08).

### 3.2 Physician‐perceived barriers to patient CCT referral

Physicians described several perceived barriers to referring patients to trials. These fell into either *patient barriers, clinical barriers, physician barriers* and/or *structural barriers*.

First, physicians cited *patient barriers*, such as the patient's age, their distrust of trials, potentially limited patient resources and their lack of self‐advocacy. Additionally, they highlighted the nature of phase 1 CCTs as a patient barrier: ‘[The] main obstacle is that it is a phase one trial. Sometimes patients have already gone through treatment and aren't sure they want to keep going’ (C12). Others discussed barriers due to patient mistrust of clinical trials generally: ‘Overall, it is difficult to enrol patients on clinical trials because of either access difficulties for the patient, or resource difficulties for the patient, or sometimes even patients’ mistrust of the trial in general’ (C14). However, patient mistrust may stem from their safety concerns. As one physician described:

When you're trying to demonstrate safety, I think that's the one thing that's causing people to be concerned about the COVID vaccines is that they're worried that there's going to be side effects that come out long term because the trials were quick in terms of what we typically would see. So, I do think that would be something that people would be concerned about. (C07)

Physicians also discussed *clinical barriers*, most often having to do with the type of practice they were in (e.g., private practice or academic medicine). For instance, some discussed clinical barriers regarding the practical aspects of working in private practice, such as referring patients, or revenue, away from their own practice: ‘Being in private practice, we obviously cannot refer all of our patients in the scope of our practice away, or we'd have no business’ (C01). Physicians also highlighted that ‘the academic institution gets reimbursed for clinical trials, but the physicians that referred them do not. This impedes early referral to trials. Especially when they have to do a lot of paperwork [or] data management’ (C06).

Physicians discussed their own *physician barriers*, such as a lack of knowledge about ongoing trials: ‘If I knew of a clinical trial, I would like to understand it first, so that I could better explain it to the patient’ (C06). However, trial information should be communicated in a way that can be readily understood by physicians, ‘because if I have a hard time understanding it, they're [the patient] going to have a real hard time understanding it’ (C06). Physicians also expressed wanting more knowledge of ‘what the most promising trials are and to be given a limited number to focus on. I would want someone from the trial to speak to us about all that they are doing’ (C02).

Similar to their practice setting (i.e., private or academic) being a possible clinical barrier, this was also at times described as a *physician barrier* (e.g., impacting whether private practice physicians qualify to refer patients to some trials; lack of communication or education about ongoing trials from research staff). For example, one physician said, ‘Well, I am private practitioner, and so I have limited ability in order to participate in clinical trials. And my knowledge as a trial progresses is going to be limited because of that as well’ (C08).

Finally, physicians discussed *structural barriers* that exist for patients, such as transportation, logistics, and distance. Travel distance and/or driving requirements impact the likelihood that patients will participate in a clinical trial: ‘They just say, “Doc, I'm not driving”.… A lot of patients have the mentality that, “I want to get treated close to my home”’ (C06). This is impacted not just by patients’ preferences but by patients’ ability to drive (e.g., ‘elderly patients specifically tend to be discouraged if clinical trials take place at a distance from them, but I have social workers who help out with this’ C01). Physicians also described time as a structural barrier impacting CCT participation: ‘The patient's more likely to show up once a month than every day in a week, so, I would say that the timing of the treatments would be important’ (C07). Additional structural barriers included ‘getting people there or bed availability or insurance issues.… It just takes forever to get it to happen sometimes’ (C15).

### 3.3 People with HNC suggestions for increasing CCT enrolment

People with HNC recommended increasing clinical trial enrolment by recruiting patients through *educating patients and highlighting the benefits of participating*, *using interpersonal methods*, and *using mediated communication channels* (Table 3).

**Table 3:** Suggestions for increasing CCT enrollment.

Categories and subcategories	Description
Suggestions from people with HNC	
Educate patients and highlight the benefits of participating	Offer assurance about trial safety, provide information about the trial to patients, and use honesty (i.e., develop patient trust).
Use interpersonal methods of communication	Recruit patients through referrals made by research staff or individual clinicians, including patients' clinicians (i.e., utilize existing doctor–patient relationships). Preferences for the type of referring clinician (e.g., oncologist, ENT) were individualized (e.g., who they see most often, have a good clinical relationship with, etc.)
Use mediated communication channels	Advertise in clinics, on the radio, television, social media and among patient support groups.
Suggestions from physicians	
Improve patient education	Promote informed patient decision‐making by providing education about CCTs, highlighting the benefits of CCTs (e.g., discovering new treatments).
Better doctor–patient communication	Encourage patients to join clinical trials, use communication to develop trust and rapport with patients, and make patients feel comfortable.
Have research staff at referring sites	To screen patients for eligibility, and reduce the burden of clinical staff in other roles.
Improve the dissemination of trial information	Through community outreach, electronic databases, and provide clinicians with regular trial information updates
Improve networking between trial doctors and referring doctors	Maintain an ongoing relationship/partnership, particularly among physicians in private practice and academic medicine.

Abbreviations: CCT, cancer clinical trial; ENT, ear, nose and throat; HNC, Head and neck cancer.

People with HNC suggested recruiting by *educating patients and highlighting the benefits of participating*. At times, this included offering assurance that the trial is safe and viewing physicians as being honest about clinical trial information (e.g., possible risks). A participant suggested to ‘assure people that, “Although it is a trial, we've taken the steps as best as possible to make it as safe as possible”. And be honest’ (P17).

People with HNC also preferred *using interpersonal methods* if they were to be recruited to CCTs, such as by individual physicians or research staff, with most wanting to be referred by their own physicians: ‘I just don't think you're going to get a whole lot of participation unless you go through physicians… I don't think there's any other way because there's a relationship already established between a physician and the patient’ (P07). However, the type of physician they thought should provide the referral varied by participant and may be influenced by where they are in the cancer continuum (e.g., during active treatment, they may want to be referred by their oncology specialists, whereas those in surveillance may want to be referred by their ENT doctor or primary care doctor). For example, a participant suggested recruiting through ‘physical therapists, speech and swallow therapists, occupational therapists, any of those services that you would typically do after you've had your first treatment’ (P10). This may also be influenced by which clinician that patients feel is most knowledgeable about their cancer history.

Others wanted to be invited to participate in a trial by physicians they feel are most knowledgeable about the trial (e.g., physicians who are investigators on the trial), not necessarily those most knowledgeable about their personal cancer history. One participant explained that this would be the best person to invite them to participate in the trial because ‘I would feel that they had very much more specific knowledge about the trial than my own doctor would’ (P28). Others said that they preferred to be invited by trial staff ‘because they are familiar with what they are doing… [but] I would probably want to run it by my oncologist’ (P06).

Finally, people with HNC suggested *using mediated communication channels* to recruit, such as using advertisements in clinics, on the radio, television, or social media, as well as patient support groups:

There's a huge group on Facebook for head and neck cancers, and that's worldwide, [so] definitely [recommend] advertising there. But even on a smaller scale, like I work with a local speech and swallow therapist who specializes in head and neck cancer patients, so even talking to them about talking to their patients or having flyers there. (P10)

### 3.4 Physician recommendations to improve CCT enrollment

Physicians had several recommendations for improving cancer clinical trial referrals. They suggested improving *patient education, using better doctor–patient communication*, *having research staff at referring sites, improving the dissemination of trial information, improving networking between trial and referring physicians* and *having local clinics as satellite clinics*.

Physicians suggested *improving patient education* about ongoing trials by discussing educational information about trials in a way that may elicit a more positive reaction from patients:

I think how they react is totally dependent on how you present the information. Because they don't know anything about the trial and I think [how] they will react will be a reflection of how you presented the information and your responsibilities to present it honestly. (C02)

Physicians also discussed the importance of patient education so that patients can make an informed decision about participating or not (e.g., ‘Patients are getting smarter. We just have to take advantage of that and keep educating the patients about the importance of clinical trials’ C04; ‘People are smart enough to make decisions, but you have the responsibility of educating them’ C02). One way that physicians may do so is by highlighting to patients that ‘ultimately, the things that we develop from a treatment standpoint, any current medicine always stem from trials’ (C07).

They also suggested that *using better doctor–patient communication* could improve trial referrals, including ‘encourage[ing] the patient to go and evaluate being part of the trial’ (C03). Improving doctor–patient communication also included communicating to develop trust and rapport:

I think a lot has to do with the comfort level with our staff, and that is one of our hallmarks in our practice, is that our patients are extremely loyal to our practice. So, with the confidence and trust they have in us, that would be something that would extend into the clinical trial. (C05)

Physicians suggested *having research staff at referring sites*, which may address barriers related to a lack of clinician knowledge about ongoing trials. This may also help to increase enrollment by having the resources (i.e., research staff) to screen patient health data for eligibility at clinician offices:

We have a research program [and] have a couple of research nurses in each office… So, research nurses, if we have a patient that we're screening, then if we don't have a trial for them, but [another clinic] has a trial, then we can inform the patient there's a trial [nearby], are you willing to travel? (C04)

Similarly, another clinician said:

We have a patient data manager who's full‐time with us. They will look at the eligibility criteria for the stuff [trials] we have, and then our nurses are pretty good [too]. If we know the clinical trial and we have it in front of us, it's [easier]. (C06)

Further, they advised *improving the dissemination of trial information* through community outreach or electronic databases. A physician suggested having ‘a broad span in terms of community outreach. Make sure that the doctors in the community, including primary care and ENTs, are aware of what is going on. Also, make sure the information is well laid out on the website and has reliable contact information’ (C07). This also included providing regular updates on available trials: ‘I think if we got an email saying ‘update’ or some sort of a link … that we could connect to on a regular basis to get an updated list of what [trials are] available’ (C10).

Physicians also recommended *improving networking between trial doctors and referring doctors* through partnerships or professional organizations. The importance of maintaining ongoing relationships and communication through physician networks was highlighted: ‘[Trial staff] can also tell referring physicians directly about the Phase I trial, ask to keep them up to date, and let them know if there are any patients that they think would be eligible’ (C06).

Networking was also important for private practice physicians, particularly with universities as many clinical trials take place at academic research institutes: ‘It's the professional association that exists between the researchers and those private practice physicians that are important because that's the basis for the patient's trust’ (C05). Not only was this seen as important for increasing CCT referrals, but also for co‐managing patient care:

If it was more of a partnership between [the university] and our practice, I think we could be more involved and get more patients in there… Because if a patient is on a clinical trial at [the university] and they're not feeling well, they can be in our office in five or 10 or 15 minutes. So, I think if it was some sort of a coordinated effort … to co‐manage the patient, I think that would be much more effective. (C10)

Finally, physicians suggested *having local clinics as satellite clinics* to make access more convenient for trial participants: ‘I don't know if there's a way of becoming a satellite for clinical trials at [the university], but I think that would be better for the patients if they don't have to leave their [community]’ (C10).

In summary, people with HNC and community physicians treating patients with HNC identified similar and disparate factors related to current barriers and future suggestions for CCT recruitment (Table 4). Both groups identified patient concerns about treatment efficacy and systematic/structural barriers as the current main inhibitors of CCT recruitment. Additionally, both groups suggested that CCTs employ more in‐depth patient education, enhance communication between current doctors and patients about CCTs, and have CCT staff aid community physicians in recruitment activities.

**Table 4:** Barriers and suggestions identified by groups.

Barriers and suggestions	Identified by…	
Barriers to patient recruitment/physician referrals	People with HNC	Physicians
Patient concerns about side effects and safety	X	
Patient lack of knowledge about CCTs	X	
Patient concerns about efficacy	X	X
Systemic/environmental/structural pt. barriers (travel, distance, cost, etc.)	X	X
Patient mistrust of vaccines or CCTs^a^		X
Type of physicians' clinical practice (private vs. academic)		X
Physician lack of knowledge about ongoing trials		X
Suggestions to improve recruitment/referrals		
Educate patients and highlight the benefits of CCT participation.	X	X
Improve doctor–patient communication/recruit through clinicians.	X	X
Use the trial staff to help recruit in clinics/provide information to patients.	X	X
Use mediated communication channels (e.g., social media).	X	
Improve dissemination of trial information to clinicians.		X
Improve networking between trial doctors and referring doctors.		X

Abbreviations: CCT, cancer clinical trial; HNC, head and neck cancer.

^a^ Although some patients expressed scepticism towards clinical trials, this was mostly attributed to patients' concerns about safety/potential side effects, limited knowledge about CCTs or concerns regarding the efficacy of the CCT treatment.

## 4 DISCUSSION

In this study, we examined the barriers to participation or referral, as well as methods of improving recruitment from both the person with HNC and community physician perspectives in preparation for a first‐in‐human Phase I therapeutic CCT for recurrent HNC. We have three main findings. First, overall themes about patient barriers to participating in CCTs reported by racial and ethnically diverse people with HNC and physicians are consistent with the literature.^15^, ^16^ Second, many of the thematic barriers can be addressed with widely suggested, but rarely implemented, collaboration between patients, community physicians, CCT researchers and trial sponsors.^15^, ^29^ Third, the required focus on and adaptation of recruitment strategies needed to conduct interviews with underrepresented people with HNC (African American, Hispanic and rural) reflect the needed focus to recruit and retain minoritized CCT participants. In summary, our findings support the American Society of Clinical Oncology and Association of Community Cancer Centers 2022 Joint Research Statement that urges institutions, researchers and sponsors to account for the needs of a broader population so that created cancer treatments are applicable to the diversity of patient populations.^29^

Consistent with prior research on barriers to CCT recruitment and referral,^16^, ^17^ multicultural people with HNC and community physicians in Florida stated patients' most pressing barriers to trial participation were lack of knowledge, concerns about side effects and efficacy, desire to receive information about the trial from a trusted source, and structural barriers (i.e., financial and travel distance).^15^ The consistency between prior research among patients deciding about a trial and our study among people with HNC who were not currently considering a trial for themselves adds evidence in support of the main thematic barriers. Similar to patients who participate in Phase I trials focusing on hope for a treatment benefit,^17^ people with HNC in our study focused their comments on the safety and efficacy of the treatment as a deciding factor and suggested recruitment efforts focus on benefits to the participant. Especially considering that our participants received the explanation that a Phase I study ‘will be among a small number of participants to make sure the vaccine is safe’, consent practices for Phase I CCTs may need to adapt to mitigate therapeutic misconceptions.^30^

Thematic barriers identified including lack of physician understanding of specific trials and desire for trust with the research team, participant desires to receive information about the trial from a trusted source, participant financial constraints and increased travel distances can be addressed by co‐design of CCT procedures. For example, Missouri Baptist Medical Center strategically increased collaboration with community physicians to increase CCT participation by offering oncological expertise to support community physicians, offering training for community clinical staff and providing community clinic support specific to CCTs.^31^ The strategies specifically aimed at CCT support addressed themes of trust and travel distances by incorporating community physicians into academic infrastructure to give them access to trials and administering trial activities in community settings in collaboration with the community physicians. Alternatively, the Ohio State University Comprehensive Cancer Center addressed participant lack of knowledge by creating community‐based coalitions to address the community's educational needs.^31^ Other recommended options include providing transportation and childcare for participants to ease the burden of participation.^32^, ^33^

The additional efforts needed to recruit minoritized people with HNC to participate in our interviews mirror the additional efforts needed to recruit minoritized CCT participants.^32^ While our traditional recruitment efforts (advertisements in multiple venues and flyers distributed throughout the community) recruited participants, they all identified as non‐Hispanic White. Like central themes for recruiting minoritized participants into CCTs,^32^ personal efforts from a trusted source were needed. Our efforts of having an HNC surgeon and known contact call participants may be unreasonable for large CCTs and are unevaluated for CCTs. However, evidence‐based strategies to increase recruitment of minoritized populations into CCTs include involving patient navigators for CCTs as well as cancer care, community engagement in education and recruitment, culturally tailored videos with racially concordant video participants, and racially concordant study coordinators.^29^, ^34^ Notably, multilevel and flexible approaches may be most successful.^11^, ^35^

Limitations of our study included that many of the people with HNC had little knowledge about clinical trials, influencing the depth of their responses. However, this limited knowledge is reflective of the population and needs to be addressed with culturally tailored educational efforts during recruitment and consent processes.^31^, ^32^, ^34^ Because we intentionally examined an HNC Phase I trial, our findings may be less generalizable to other types of trials. In addition, it is worth noting that the conversations with the participants were directed towards our research questions with an interview guide that may have influenced the responses of participants. Strengths of our study included the use of an interdisciplinary team that facilitated the creation of a rigorous interview guide and supported the recruitment of a racially and ethnically diverse sample of people with HNC.

Recruitment of participants, especially participants identifying with underrepresented population subgroups, continues to be a challenge to CCTs.^29^, ^34^ While clear solutions remain relatively allusive, understanding local population barriers to participation and referral before recruitment such as by performing a qualitative needs assessment as shown in this paper, may aid the selection of the most relevant evidence‐based strategies. For example, in our population, CCT recruitment strategies will likely be best received if directed towards improving communication between the community physicians and the academic centre, creating strategies to specifically address systematic barriers, and providing educational opportunities for potential participants and community physicians. Our findings are in line with the principles proposed by the American Society of Clinical Oncology and Association of Community Cancer Centers and other ongoing research into strategies to increase racial and ethnic diversity in CCTs.

## CONFLICT OF INTEREST STATEMENT

The authors declare no conflict of interest.

## Data Availability

The data that support the findings of this study are available on request from the corresponding author. The data are not publicly available due to privacy or ethical restrictions.
